# Gene expression profiling of the tumor microenvironment during breast cancer progression

**DOI:** 10.1186/bcr2222

**Published:** 2009-02-02

**Authors:** Xiao-Jun Ma, Sonika Dahiya, Elizabeth Richardson, Mark Erlander, Dennis C Sgroi

**Affiliations:** 1 bioTheranostics, Inc., 11025 Roselle Street, Suite 200, San Diego, CA 92121, USA; 2 Molecular Pathology Unit and Center for Cancer Research, Massachusetts General Hospital, 149 13th Street, Charlestown, MA 02129, USA

## Abstract

**Introduction:**

The importance of the tumor microenvironment in breast cancer has been increasingly recognized. Critical molecular changes in the tumor stroma accompanying cancer progression, however, remain largely unknown. We conducted a comparative analysis of global gene expression changes in the stromal and epithelial compartments during breast cancer progression from normal to preinvasive to invasive ductal carcinoma.

**Methods:**

We combined laser capture microdissection and gene expression microarrays to analyze 14 patient-matched normal epithelium, normal stroma, tumor epithelium and tumor-associated stroma specimens. Differential gene expression and gene ontology analyses were performed.

**Results:**

Tumor-associated stroma undergoes extensive gene expression changes during cancer progression, to a similar extent as that seen in the malignant epithelium. Highly upregulated genes in the tumor-associated stroma include constituents of the extracellular matrix and matrix metalloproteases, and cell-cycle-related genes. Decreased expression of cytoplasmic ribosomal proteins and increased expression of mitochondrial ribosomal proteins were observed in both the tumor epithelium and the stroma. The transition from preinvasive to invasive growth was accompanied by increased expression of several matrix metalloproteases (MMP2, MMP11 and MMP14). Furthermore, as observed in malignant epithelium, a gene expression signature of histological tumor grade also exists in the stroma, with high-grade tumors associated with increased expression of genes involved in immune response.

**Conclusions:**

Our results suggest that the tumor microenvironment participates in tumorigenesis even before tumor cells invade into stroma, and that it may play important roles in the transition from preinvasive to invasive growth. The immune cells in the tumor stroma may be exploited by the malignant epithelial cells in high-grade tumors for aggressive invasive growth.

## Introduction

The tumor microenvironment or the stroma hosting the malignant breast epithelial cells is comprised of multiple cell types, including fibroblasts, myoepithelial cells, endothelial cells and various immune cells [1-4]. One prevailing view is that tumor-associated stroma is activated by the malignant epithelial cells to foster tumor growth – for example, by secreting growth factors, increasing angiogenesis, and facilitating cell migration, ultimately resulting in metastasis to remote organ sites [3]. For example, two chemokines (chemokine (C-X-C motif) ligand (CXCL) 12 and CXCL14) that bind to tumor epithelial cells to promote proliferation, migration and invasion have recently been shown to be overexpressed by the activated tumor fibroblasts and myoepithelial cells [5-7]. Genes involved in tumor-microenvironment interactions may therefore provide novel targets for diagnostic development and therapeutic intervention. Our understanding of the interactions between epithelial and stromal components of breast cancer, however, remains limited at the molecular level. Using the serial analysis of gene expression technique, Allinen and coworkers performed the first systematic profiling of the various stromal cell types isolated via cell-type-specific cell surface markers and magnetic beads [7]. They demonstrated gene expression alterations in all cell types within the tumor microenvironment accompanying progression from normal breast tissue to ductal carcinoma *in situ *(DCIS) to invasive ducal carcinoma (IDC) [8], providing evidence that these cell types all participate in tumorigenesis.

Using laser capture microdissection (LCM), we previously performed gene expression analysis of the epithelial compartment of the malignant lesions during breast cancer progression. We discovered that most of the gene expression changes take place prior to local invasion (even in atypical ductal hyperplasia) and that there are no major changes in gene expression accompanying the *in situ *to invasive growth transition [9]. In the present article we extend this analysis to the tumor stromal microenvironment and demonstrate that, like the tumor epithelium, the tumor stromal microenvironment undergoes extensive gene expression alterations even at the preinvasive stage of DCIS, supporting the view that cell-cell communication via paracrine mechanisms between the two compartments plays an important role in tumor progression.

## Materials and methods

### Clinical specimen

All breast cancer specimens were fresh-frozen biopsies obtained from the Massachusetts General Hospital between 1998 and 2001. The diagnostic criteria and tumor grading were described previously [9]. Patient and tumor characteristics of the 14 tumor specimens in this study are presented in Table 1. Patients were selected in which patient-matched normal and tumor samples were available and the normal breast lobules did not show fibrocystic change. The research was deemed exempt from informed consent as the samples are unidentifiable to the research team. The study was approved by the Massachusetts General Hospital human research committee in accordance with National Institutes of Health human research study guidelines.

**Table 1:** Patient and tumor characteristics of samples in the study

Patient number	Age (years)	Grade	Estrogen receptor	Progesterone receptor	Her-2	Size	Nodal status	Tumor type
44	28	III	Positive	Positive	Negative	1	Negative	Ductal
45	36	I	Positive	Positive	Negative	N/A	Negative	Ductal
79	54	I	Positive	Positive	Negative	2.1	Positive	Ductal
96	31	III	Negative	Negative	Negative	3.7	Negative	Ductal
102	55	I	Positive	Negative	Negative	5.2	Positive	Ductal
121	45	II	Positive	Positive	Positive	1.5	Positive	Ductal
131	37	II	Positive	Positive	Positive	1.5	Positive	Ductal
133	44	III	Negative	Negative	Positive	1.5	Positive	Ductal
148	42	II	Positive	Positive	Negative	1.9	Positive	Ductal
153	46	I	Positive	Positive	ND	N/A	Positive	Ductal
169	34	II	Positive	Positive	Negative	2.6	Positive	Ductal
178	43	III	Positive	Positive	Positive	2.8	Positive	Ductal
179	37	III	Negative	Negative	Positive	1.5	Positive	Ductal
180	46	I	Positive	Positive	Negative	1.9	Positive	Ductal

ND, not determined; N/A, not available.

### Laser capture microdissection, RNA extraction and microarray analysis

**Figure 1:**
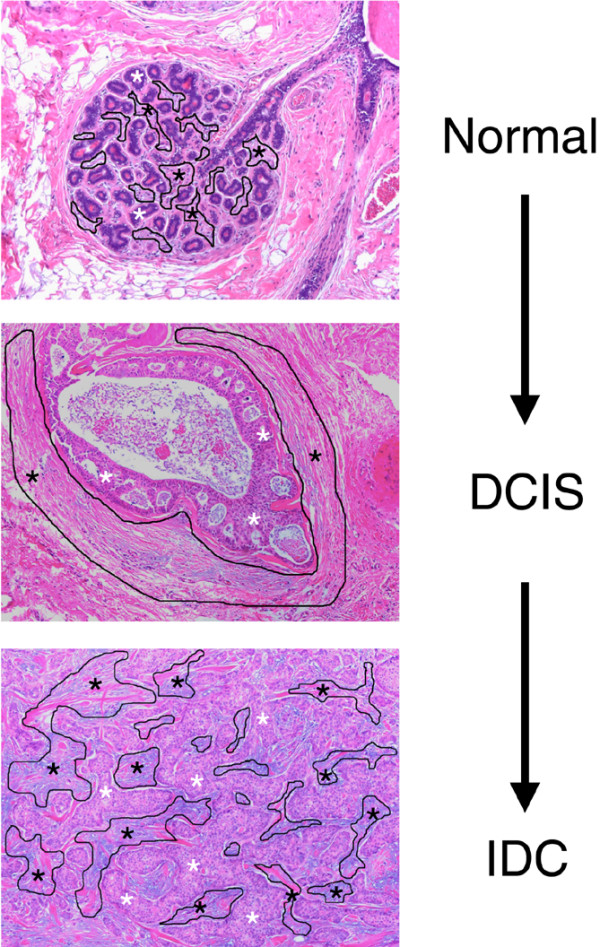
Laser capture microdissection experimental design. Example of the tumor microenvironment compartments targeted by laser capture microdissection: epithelial (white asterisk) and stromal (black outlined areas with black asterisk) compartments of the normal terminal ductal lobular unit, of ductal carcinoma *in situ *(DCIS) and of invasive ductal carcinoma (IDC).

Highly enriched populations of patient-matched normal or malignant epithelial cells and of normal stroma or tumor-associated stroma from the different stages of breast cancer progression were procured by LCM using a PixCell IIe system (Molecular Devices, Mountain View, CA, USA) as previously described [9]. Enrichment for cells of interest was verified by microscopic examination of the LCM cap after microdissection. The microdissected normal stromal compartment consisted of the intralobular, rather than the extralobular, stromal compartment of normal breast tissue that was a minimum 0.3 cm from any premalignant or malignant lesion (Figure 1). The DCIS-associated stroma (DCIS-S) consisted of a 25 μm rim of cells that surrounded the DCIS; for cases in which synchronous DCIS and IDC were present, the DCIS-S was obtained from areas of DCIS that were at least 0.3 cm from the invasive component. The IDC-associated stroma (IDC-S) consists of stromal cells predominantly within the invasive tumor mass.

Total RNA was isolated from captured cells using the Picopure™ RNA isolation kit (Molecular Devices), amplified by T7 RNA amplification (RiboAmp™; Molecular Devices), labeled and hybridized to the whole genome array U133X3P (3'-biased design) according to the manufacturer's instructions (Affymetrix, Santa Clara, CA, USA). The hybridized microarrays were then washed, stained and scanned as per the manufacturer's protocols (Affymetrix).

### Data analysis

Raw data from the U133X3P arrays were processed using the Bioconductor rma package with default parameters for background correction, quantile normalization and signal summation [10,11]. Differential gene expression analyses were performed using linear regression models in the limma package [12]. For comparing normal and tumor samples, we used the patient identification as a blocking variable. For tumor grade comparison, we used the tumor stage (*in situ *or invasive) as the blocking variable. Statistical significance was corrected for multiple testing using the Benjamini-Hochberg procedure [13]. All procedures were performed in the R statistical environment [14]. For gene ontology analysis, ranked gene lists were first generated according to the moderated *t *statistics from linear models and then examined for enriched ontology terms using the Gene Set Enrichment Analysis software [15]. The data discussed in this publication have been deposited in the NCBI Gene Expression Omnibus [16] and are accessible [GEO:GSE14548] [17].

### Quantitative real-time PCR and immunohistochemistry

TaqMan™ real-time PCR was performed on amplified RNA used for microarray analysis as previously described [9]. Briefly, amplified RNA was converted to double-stranded cDNA, and the cDNA was quantitated with PicoGreen (Molecular Probes, Eugene, OR, USA) using a spectrofluorometer (Molecular Devices). Each gene was analyzed in triplicate in a 96-well plate using ABI 7900 HT (Applied Biosystems, Foster City, CA., USA).

For each gene, the sequences of the PCR primer pairs and the fluorogenic MGB or TAMRA probe (5' to 3'), respectively, are as follows: ESR1, ATGATCAACTGGGCGAAGA, GGTGGACCTGATCATGGA and VIC-TGCCAGGCTTTGTGGA; RRM2, CCTTTAACCAGCACAGCCAGTT, TTATTTGTTTGTAAAGTGCCAGGTTT and VIC-TGCAGCCTCACTGCTTCAACGCA-TAMRA; gremlin 1 (GREM1), ACGGCAAAGAATTATATAGACTATGAGGTA, TTTTATGAGACTATCAACTCCCCTTTC and VIC-CTTGCTGTGTAGGAGGA; and WNT inhibitory factor 1(WIF1), CACTGTGGTAGTGGCATTTAAACAATA, GCCAATGCAAAAAGTTCATACATT and VIC-TTCTAAACACAATGAAATAGGGA.

Estrogen receptor and progesterone receptor immunohistochemistry staining was performed as previously described, using the rabbit monoclonal antibody (SP1) from Lab Vision (Fremont, CA, USA) for the estrogen receptor (1:50 dilution) and using the mouse monoclonal antibody (PgR 636) from Dako (Carpinteria, CA, USA) for the progesterone receptor (1:50 dilution) [18].

## Results

### Experimental design

The present study included 14 patients with primary ductal breast cancer (Table 1). These patients were primarily estrogen receptor positive (78.6%), lymph node positive (78.6%), and premenopausal (mean age 41 years). We used LCM to isolate the epithelial and stroma compartments separately from each of the 14 fresh-frozen biopsies. In the epithelial compartment, we captured normal and malignant epithelium from DCIS and/or IDC. In the stromal compartment, we captured normal stroma at least 3 mm from the malignant lesion and the DCIS-S and/or IDC-S whenever possible. An example of the microdissected compartments is shown in Figure 1. As shown in Table 2, in the epithelial compartment four cases had all three stages (normal breast epithelium, DCIS, and IDC) available, five cases had normal breast epithelium and IDC only, and five cases had normal breast epithelium and DCIS only; in the stroma, six cases had all three stages available, five cases had normal stromal compartment and DCIS-S, and three cases had the normal stromal compartment and IDC-S. RNA was isolated from the captured cells and interrogated with the Affymetrix whole-genome array U133X3P.

**Table 2:** Laser capture microdissection of 14 primary breast cancer patients

	Tumor			Stroma		
	
Patient	Normal	*In situ*	Invasive	Normal	*In situ*	Invasive
44	x	x	x	x	x	x
45	x		x	x	x	x
79	x		x	x	x	x
96	x	x	x	x	x	x
102	x	x	x	x	x	x
121	x	x	x	x	x	x
131	x	x		x	x	
133	x	x		x	x	
148	x		x	x		x
153	x		x	x		x
169	x		x	x		x
178	x	x		x	x	
179	x	x		x	x	
180	x	x		x	x	

x, component captured.

### Gene expression changes in the stromal and epithelial compartments during breast cancer progression

We compared the gene expression patterns of the tumor epithelium and stroma at each stage of progression (DCIS or IDC) with their respective normal state using the limma (linear models of microarrays) software package [12]. The resulting *P *values for differential gene expression in each pair-wise comparison were adjusted for multiple testing [13], and the genes with a significant adjusted *P *value (*P *<0.05) were extracted.

**Figure 2:**
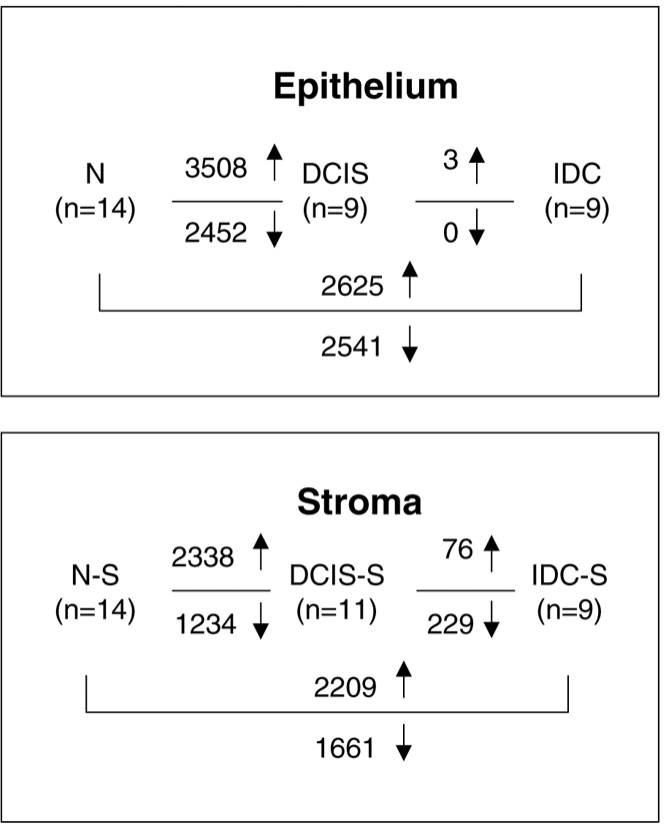
Comparative analysis of gene expression changes in tumor and stroma. Gene expression changes in normal breast epithelium (N), ductal carcinoma *in situ *(DCIS), invasive ductal carcinoma (IDC), normal stromal compartment (N-S), ductal carcinoma *in situ*-associated stroma (DCIS-S) and invasive ductal carcinoma-associated stroma (IDC-S). ↑, upregulated genes; ↓, downregulated genes.

**Figure 3:**
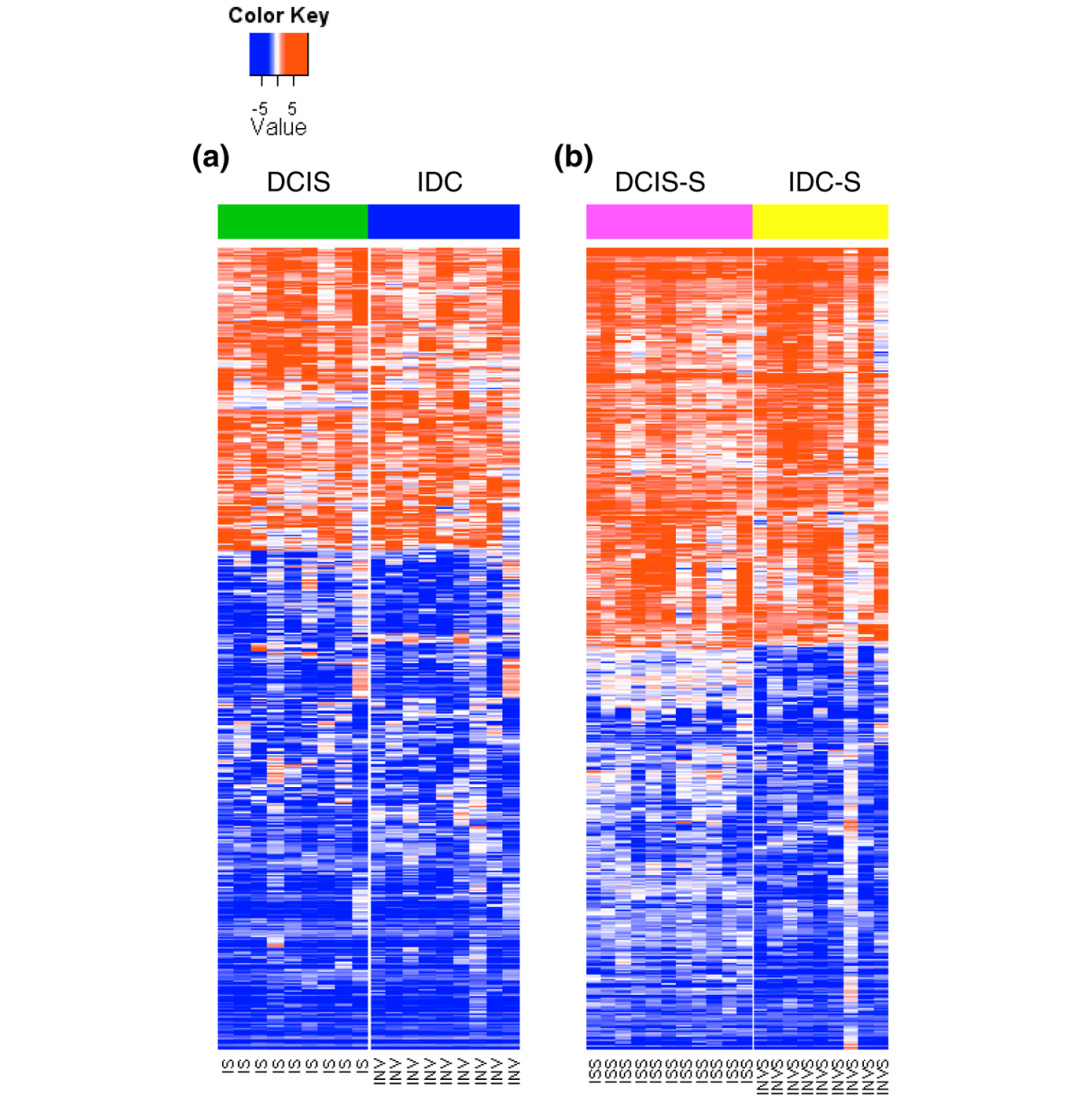
Heatmap of expression patterns of ductal carcinoma *in situ*-associated and invasive ductal carcinoma-associated genes. **(a) **Heatmap of 849 genes with >3-fold differential expression in either ductal carcinoma *in situ *(DCIS) versus normal breast or invasive ductal carcinoma (IDC) versus normal breast in the epithelium. **(b) **Heatmap of 557 genes with >3-fold differential expression in either ductal carcinoma *in situ*-associated stroma (DCIS-S) versus normal stromal compartment or invasive ductal carcinoma-associated stroma (IDC-S) versus normal stromal compartment. Data shown are log_2_(fold change) relative to the average expression in normal controls (normal breast epithelium or normal stromal compartment). In each heatmap, genes (rows) are hierarchically clustered using 1 – Pearson correlation as the distance metric. IS, ductal carcinoma in situ; INV, invasive ductal carcinoma; ISS, ductal carcinoma in situ-associated stroma; INVS, invasive ductal carcinoma-associated stroma.

The DCIS and IDC stages were each associated with thousands of gene expression alterations relative to their respective normal state in both the tumor epithelium and the stroma (Figure 2). Furthermore, within each compartment, the expression patterns of DCIS-associated and IDC-associated genes were highly similar to each other (Figure 3).

To gain an overview of the biological processes in which these differentially expressed genes are involved, we performed gene set enrichment analysis [19] using the gene ontology database [20]. Table 3 presents the top 20 gene ontology terms significantly enriched within genes upregulated in the invasive stage in the epithelium and the stroma. In the epithelium, the genes were dominated by those associated with the cell cycle (mitosis in particular). In the stroma, the genes prominently featured the components of the extracellular matrix and the matrix metalloproteases responsible for remodeling the extracellular matrix. Additionally, the stromal genes also included those related to the cell cycle, indicating increased proliferation as a common feature in both the tumor epithelium and the stroma.

**Table 3:** Top 20 gene ontology terms enriched in tumor epithelium and stroma

Name	Size (number of genes)	Normalized enrichment score	False discovery rate *q *value
Epithelium			
SPINDLE	39	2.33	0
CHROMOSOME_SEGREGATION	28	2.15	0
CELL_CYCLE_PROCESS	180	2.12	0
MICROTUBULE_CYTOSKELETON_ORGANIZATION_AND_BIOGENESIS	34	2.11	0
CHROMOSOME__PERICENTRIC_REGION	27	2.11	0
MICROTUBULE_CYTOSKELETON	142	2.11	0
PROTEASOME_COMPLEX	22	2.09	1.40 × 10^-4^
CONDENSED_CHROMOSOME	30	2.06	2.48 × 10^-4^
M_PHASE	105	2.06	2.20 × 10^-4^
NUCLEAR_ENVELOPE	71	2.05	1.98 × 10^-4^
CELL_CYCLE_PHASE	157	2.05	1.80 × 10^-4^
M_PHASE_OF_MITOTIC_CELL_CYCLE	78	2.04	2.49 × 10^-4^
CHROMOSOME	115	2.03	2.30 × 10^-4^
CYTOSKELETAL_PART	221	2.03	2.14 × 10^-4^
MITOSIS	75	2.02	2.66 × 10^-4^
MICROTUBULE	32	1.99	2.49 × 10^-4^
MITOTIC_CELL_CYCLE	139	1.99	2.35 × 10^-4^
CELL_CYCLE_CHECKPOINT_GO_0000075	45	1.98	2.21 × 10^-4^
SPINDLE_MICROTUBULE	16	1.97	2.63 × 10^-4^
DNA_REPAIR	120	1.94	6.01 × 10^-4^
*STRUCTURAL_CONSTITUENT_OF_RIBOSOME*	*74*	*-3.09*	*0*
Stroma			
EXTRACELLULAR_MATRIX_STRUCTURAL_CONSTITUENT	25	2.12	0
COLLAGEN	23	2.07	0.001566
METALLOENDOPEPTIDASE_ACTIVITY	26	2.06	0.001044
EXTRACELLULAR_MATRIX	94	1.99	0.001568
PROTEINACEOUS_EXTRACELLULAR_MATRIX	93	1.97	0.002923
EXTRACELLULAR_MATRIX_PART	54	1.91	0.007826
SPINDLE	39	1.89	0.008346
METALLOPEPTIDASE_ACTIVITY	45	1.82	0.027006
SKELETAL_DEVELOPMENT	99	1.80	0.032482
*STRUCTURAL_CONSTITUENT_OF_RIBOSOME*	*74*	*-3.04*	*0*

**Figure 4:**
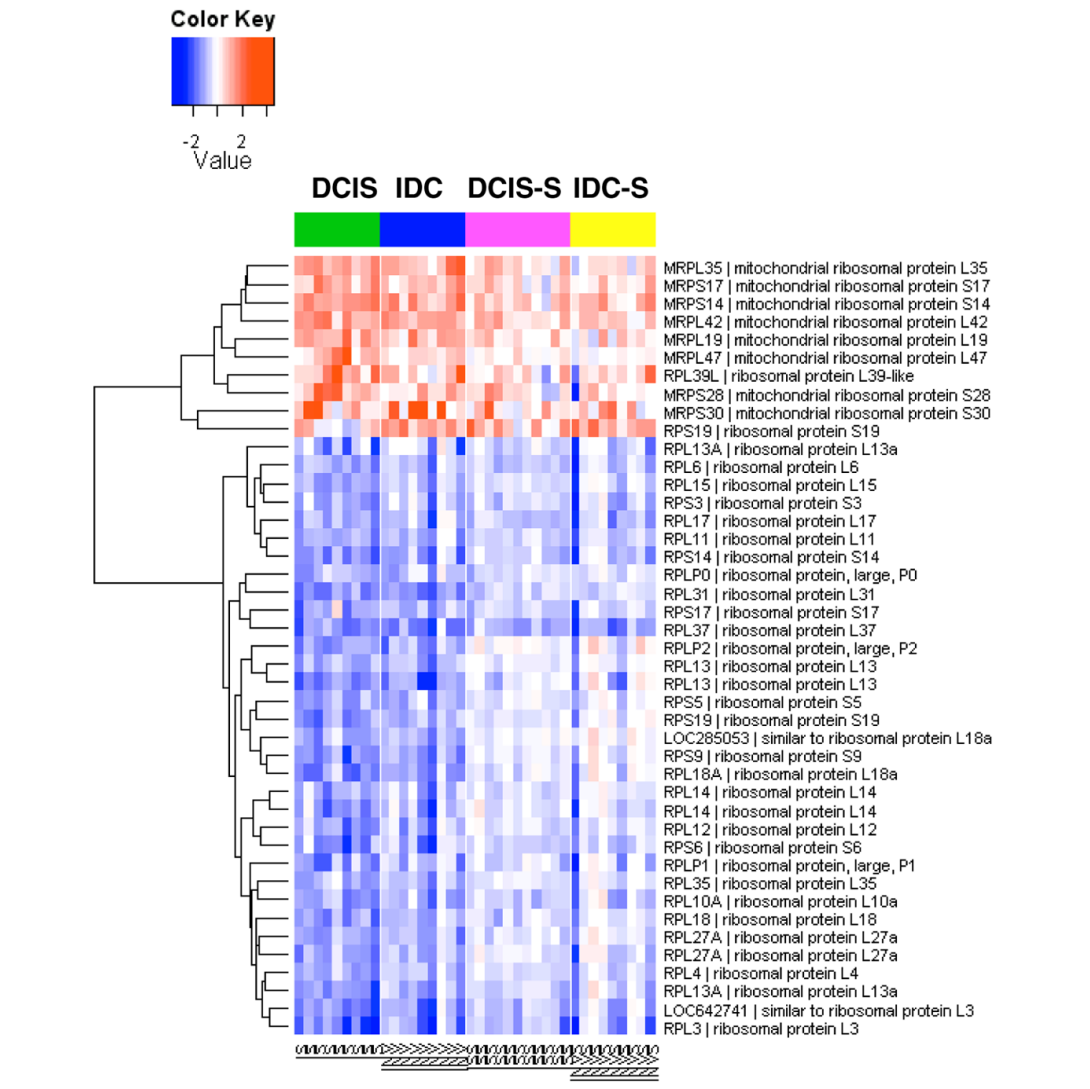
Heatmap of differential expression of ribosomal protein genes in the malignant epithelium and tumor stroma. Differential expression of ribosomal protein genes in ductal carcinoma *in situ *(DCIS), invasive ductal carcinoma (IDC), ductal carcinoma *in situ*-associated stroma (DCIS-S) and invasive ductal carcinoma-associated stroma (IDC-S). Data shown are log_2_(fold change) relative to the average expression level in the normal controls (normal breast epithelium or normal stromal compartment). Expression measurements for multiple probe sets representing the same gene were collapsed to the single representative probe set with the largest differential gene expression. All genes shown were significant at adjusted *P *< 0.05. IS, ductal carcinoma in situ; INV, invasive ductal carcinoma; ISS, ductal carcinoma in situ-associated stroma; INVS, invasive ductal carcinoma-associated stroma.

In both compartments, the single gene ontology term STRUCTURAL_CONSTITUENT_OF_RIBOSOME was significantly enriched within the downregulated genes (Table 3). To examine this further, we extracted all ribosomal protein-encoding genes that were differentially expressed between DCIS or IDC versus the normal breast in the epithelium and visualized their expression patterns in both compartments. Interestingly, there was an almost complete bipartite partitioning of these genes (Figure 4): while the downregulated genes were all those encoding for the cytoplasmic ribosomal proteins, the upregulated genes were mostly those encoding for the mitochondrial ribosomal proteins.

In addition to these global patterns, Tables 4 and 5 present the top 50 differentially expressed genes in the epithelium and the stroma, respectively. In these tables, besides the dominant features of cell-cycle-related genes in the epithelium and extracellular matrix genes in the stroma discussed earlier, we note several additional genes important in cell signaling pathways. Two antagonists of WNT receptor signaling, WIF1 and secreted frizzled-related protein 1 (SFRP1), were downregulated in both the tumor epithelium and the stroma. In addition, two members of the transforming growth factor beta superfamily, GREM1 and inhibin beta A (INHBA), showed markedly increased expression specifically in the tumor stroma (Table 5).

**Table 4:** Top 50 genes differentially expressed in tumor epithelium

Probe set	DICS	IDC	Gene description
g5174662_3p_at	5.3	4.0	S100P – S100 calcium binding protein P
g11993936_3p_s_at	4.5	3.4	CYB561 – cytochrome b-561
g7415720_3p_a_at	4.0	3.0	SCD – stearoyl-CoA desaturase (delta-9-desaturase)
Hs.75319.0.S3_3p_at	3.0	3.8	RRM2 – ribonucleotide reductase M2 polypeptide
Hs.106552.0.S3_3p_s_at	4.2	2.3	CNTNAP2 – contactin associated protein-like 2
g12803628_3p_at	3.7	2.6	HIST1H1C – histone cluster 1, H1c
g5031780_3p_at	4.0	2.3	IFI27 – interferon, alpha-inducible protein 27
Hs.180779.1.S1_3p_at	3.1	3.0	HIST1H2BD – histone cluster 1, H2bd
Hs.184572.0.S2_3p_at	2.7	3.3	CDC2 – cell division cycle 2, G1 to S and G2 to M
Hs.223025.0.S2_3p_a_at	2.6	3.4	RAB31 – RAB31, member RAS oncogene family
g12804874_3p_a_at	2.8	3.1	RRM2 – ribonucleotide reductase M2 polypeptide
Hs.239884.0.S1_3p_x_at	3.5	2.4	HIST1H2BC – histone cluster 1, H2bc
g7661973_3p_at	2.7	3.1	MELK – maternal embryonic leucine zipper kinase
g13259549_3p_at	3.2	2.4	IFI6 – interferon, alpha-inducible protein 6
g4504584_3p_at	3.5	2.0	IFIT1 – interferon-induced protein with tetratricopeptide repeats 1
Hs.155956.0.S1_3p_at	2.6	2.8	NAT1 – *N*-acetyltransferase 1 (arylamine *N*-acetyltransferase)
Hs.152677.0.S1_3p_at	3.1	2.3	DHRS2 – dehydrogenase/reductase (SDR family) member 2
Hs.239884.0.S1_3p_at	3.3	2.1	HIST1H2BC – histone cluster 1, H2bc
g5803130_3p_a_at	2.2	3.2	RAB31 – RAB31, member RAS oncogene family
g13699814_3p_s_at	2.7	2.7	CYP2B6 – cytochrome P450, family 2, subfamily B, polypeptide 6
g9963780_3p_a_at	2.2	3.2	RAB31 – RAB31, member RAS oncogene family
Hs.72472.0.A1_3p_at	2.3	2.9	-
g13477106_3p_s_at	3.3	1.8	CEACAM6 – carcinoembryonic antigen-related cell adhesion molecule 6 (nonspecific cross-reacting antigen)
Hs.133342.0.S1_3p_x_at	2.9	2.2	GPC1 – glypican 1
Hs.325335.1.S1_3p_at	2.6	2.5	CAPN13 – calpain 13
Hs.34853.0.S2_3p_at	-3.5	-4.0	ID4 – inhibitor of DNA binding 4, dominant negative helix-loop-helix protein
g3387766_3p_a_at	-3.9	-3.6	GPM6B – glycoprotein M6B
Hs.10587.0.S1_3p_at	-3.2	-4.3	DMN – desmuslin
Hs2.348883.1.S1_3p_s_at	-3.9	-3.6	FOXC1 – forkhead box C1
g4758521_3p_at	-4.9	-2.7	SPARCL1 – SPARC-like 1 (mast9, hevin)
g11037715_3p_x_at	-3.9	-3.7	ROPN1 – ropporin, rhophilin associated protein 1
g4504914_3p_at	-3.9	-3.8	KRT15 – keratin 15
Hs.82101.0.S3_3p_at	-3.7	-4.0	PHLDA1 – pleckstrin homology-like domain, family A, member 1
Hs.149356.0.S1_3p_at	-3.8	-3.9	LOC728264 – hypothetical protein LOC728264
g4506856_3p_s_at	-3.3	-4.6	CX3CL1 – chemokine (C-X3-C motif) ligand 1
g4506516_3p_at	-3.9	-4.1	RGS2 – regulator of G-protein signaling 2, 24 kDa
g7657105_3p_at	-4.2	-4.0	GABRP – gamma-aminobutyric acid (GABA) A receptor, pi
Hs.25956.0.S1_3p_at	-4.1	-4.4	SOSTDC1 – sclerostin domain containing 1
Hs.153961.2.S2_3p_at	-4.6	-3.9	BOC – Boc homolog (mouse)
g11991655_3p_at	-4.5	-4.2	C2orf40 – chromosome 2 open reading frame 40
Hs.288850.0.S1_3p_at	-4.1	-4.7	PHLDA1 – pleckstrin homology-like domain, family A, member 1
g7662650_3p_at	-5.1	-3.7	C13orf15 – chromosome 13 open reading frame 15
g4557694_3p_a_at	-4.5	-4.5	KIT – v-kit Hardy-Zuckerman 4 feline sarcoma viral oncogene homolog
Hs.127428.2.S2_3p_a_at	-4.3	-4.9	HOXA9 – homeobox A9
g6005949_3p_at	-4.4	-4.8	WIF1 – WNT inhibitory factor 1
Hs.34853.0.S3_3p_at	-4.8	-4.5	ID4 – inhibitor of DNA binding 4, dominant negative helix-loop-helix protein
g4559274_3p_a_at	-4.1	-5.3	ELF5 – E74-like factor 5 (ets domain transcription factor)
g8400731_3p_a_at	-4.4	-5.2	SFRP1 – secreted frizzled-related protein 1
g5032314_3p_a_at	-4.9	-4.9	DMD – dystrophin (muscular dystrophy, Duchenne and Becker types)
g6005714_3p_at	-4.7	-5.6	SLC6A14 – solute carrier family 6 (amino acid transporter), member 14

Ductal carcinoma *in situ *(DCIS) and invasive ductal carcinoma (IDC) data presented as log_2_(fold changes) relative to normal epithelium.

**Table 5:** Top 50 genes differentially expressed in tumor-associated stroma

Probe set	DCIS	IDC	Gene description
Hs.179729.0.S1_3p_a_at	6.5	7.0	COL10A1 – collagen, type X, alpha 1(Schmid metaphyseal chondrodysplasia)
Hs.28792.0.S1_3p_at	5.9	6.0	NA
4876385_3p_at	4.9	6.3	COL11A1 – collagen, type XI, alpha 1
g7019348_3p_at	4.9	5.7	GREM1 – gremlin 1, cysteine knot superfamily, homolog (*Xenopus laevis*)
Hs.179729.1.S1_3p_a_at	4.8	5.5	COL10A1 – collagen, type X, alpha 1(Schmid metaphyseal chondrodysplasia)
Hs.297939.3.S1_3p_at	4.6	5.1	FNDC1 – fibronectin type III domain containing 1
37892_3p_at	4.1	5.6	COL11A1 – collagen, type XI, alpha 1
Hs.41271.0.S1_3p_at	4.7	4.9	COL8A1 – collagen, type VIII, alpha 1
g4502938_3p_s_at	3.8	5.4	COL11A1 – collagen, type XI, alpha 1
g186414_3p_a_at	4.5	4.4	INHBA – inhibin, beta A
g8393842_3p_at	4.4	4.4	NOX4 – NADPH oxidase 4
Hs.288467.0.S1_3p_at	3.8	4.8	LRRC15 – leucine rich repeat containing 15
Hs.105700.0.S1_3p_a_at	4.1	4.2	SFRP4 – secreted frizzled-related protein 4
g4481752_3p_at	3.6	4.4	GJB2 – gap junction protein, beta 2, 26 kDa
g8923132_3p_at	3.6	4.3	ASPN – asporin
Hs.287820.2.A1_3p_s_at	3.8	4.1	FN1 – fibronectin 1
g8400733_3p_a_at	3.5	3.9	SFRP4 – secreted frizzled-related protein 4
g10863087_3p_a_at	3.4	3.9	GREM1 – gremlin 1, cysteine knot superfamily, homolog (*Xenopus laevis*)
g5174662_3p_at	3.4	3.4	S100P – S100 calcium binding protein P
Hs.283713.0.A1_3p_at	3.2	3.5	CTHRC1 – collagen triple helix repeat containing 1
g4502844_3p_at	3.1	3.5	CILP – cartilage intermediate layer protein, nucleotide pyrophosphohydrolase
Hs.76722.2.S1_3p_at	2.8	3.7	-
Hs.70823.0.S3_3p_at	3.3	3.2	SULF1 – sulfatase 1
g4505186_3p_at	3.3	3.0	CXCL9 – chemokine (C-X-C motif) ligand 9
Hs.101302.0.S2_3p_s_at	2.2	3.9	COL12A1 – collagen, type XII, alpha 1
g11415037_3p_at	-3.2	-3.0	SLC22A3 – solute carrier family 22 (extraneuronal monoamine transporter), member 3
Hs.325823.0.A1_3p_at	-2.7	-3.5	CD36 – CD36 molecule (thrombospondin receptor)
g4557418_3p_at	-2.7	-3.6	CD36 – CD36 molecule (thrombospondin receptor)
g4557544_3p_a_at	-2.9	-3.5	EDN3 – endothelin 3
Hs2.147313.1.S1_3p_s_at	-2.9	-3.5	CD300LG – CD300 molecule-like family member g
Hs.106283.4.S1_3p_at	-3.0	-3.4	KLHL13 – kelch-like 13 (Drosophila)
g8400731_3p_a_at	-2.6	-4.0	SFRP1 – secreted frizzled-related protein 1
Hs.250692.0.S4_3p_at	-3.0	-3.7	HLF – hepatic leukemia factor
g4826977_3p_at	-3.3	-3.5	RELN – reelin
Hs.76325.1.A1_3p_x_at	-3.0	-3.9	IGJ – immunoglobulin J polypeptide, linker protein for immunoglobulin alpha and mu polypeptides
Hs.76325.1.A1_3p_at	-3.1	-4.0	IGJ – immunoglobulin J polypeptide, linker protein for immunoglobulin alpha and mu polypeptides
g10835124_3p_a_at	-4.0	-3.1	DCX – doublecortex; lissencephaly, X-linked (doublecortin)
g7657105_3p_at	-3.2	-4.0	GABRP – gamma-aminobutyric acid A receptor, pi
g4506328_3p_at	-3.4	-3.9	PTPRZ1 – protein tyrosine phosphatase, receptor-type, Z polypeptide 1
g4758377_3p_at	-3.9	-3.4	FIGF – c-fos induced growth factor (vascular endothelial growth factor D)
g12707575_3p_at	-3.3	-4.1	OXTR – oxytocin receptor
g13518036_3p_a_at	-2.5	-4.9	MATN2 – matrilin 2
g4559274_3p_a_at	-3.9	-3.6	ELF5 – E74-like factor 5 (ets domain transcription factor)
Hs.10587.0.S1_3p_at	-2.5	-5.1	DMN – desmuslin
Hs.49696.0.A1_3p_at	-3.7	-4.0	SCARA5 – scavenger receptor class A, member 5 (putative)
g4557578_3p_at	-3.4	-4.4	FABP4 – fatty acid binding protein 4, adipocyte
g13186315_3p_a_at	-3.5	-4.3	CAPN6 – calpain 6
g11991655_3p_at	-3.2	-5.3	C2orf40 – chromosome 2 open reading frame 40
g562105_3p_a_at	-4.9	-4.5	DLK1 – delta-like 1 homolog (Drosophila)
g6005949_3p_at	-5.0	-4.8	WIF1 – WNT inhibitory factor 1

Ductal carcinoma *in situ *(DCIS) and invasive ductal carcinoma (IDC) data presented as log_2_(fold changes) relative to normal stroma.

### Stromal gene expression signature associated with tumor invasion

We next compared the gene expression patterns associated with the DCIS to IDC transition within each compartment. In the tumor epithelium, there were only three genes (POSTN, periostin; SPARC, osteoconectin; SPARCL1, SPARC-like 1) that were significantly upregulated in IDC relative to DCIS. All three genes are known to be specifically expressed in the stroma [21-23] and were indeed strongly expressed in the stroma samples in our dataset. Their apparent overexpression in IDC relative to DCIS might therefore be due to contaminating stromal cells in the procured epithelial cell populations in the IDC samples but not in DCIS samples. In the stroma, however, there were more significant changes in comparing IDC-S with DCIS-S, with 76 upregulated genes and 229 downregulated genes (Figure 2). The lack of significant changes in gene expression in the epithelium associated with the DCIS-IDC transition seen here was consistent with that in our previous study [9].

Table 6 presents the top 50 differentially expressed genes between DICS-S and IDC-S (see Additional data file 1). Among genes with increased expression in IDC-S, three matrix metalloproteases (MMP11, MMP2 and MMP14) were notable. In fact, one additional matrix metalloprotease (MMP13) had higher expression in IDC-S than in DCIS-S, with adjusted *P *= 0.06. These genes have been known to be involved in tumor invasion [3]. On the other hand, genes with decreased expression in IDC-S included many genes involved in vasculature development (for example, EMCN, FLT1, KDR, SELE, MYH11, EDNRB and PODXL), a process expected to increase in invasive cancer. This paradoxical result might reflect the decreased vascular density in the leading invasive front where we microdissected the stroma relative to the stroma surrounding DCIS.

**Table 6:** Top 50 genes differentially expressed in invasive stroma compared to in situ stroma

Probe set	Log_2_ (fold change)	Adjusted *P *value	Gene description
Hs2.434299.1.S1_3p_at	1.61	8.58 × 10^-3^	-
g13027795_3p_s_at	1.45	1.74 × 10^-2^	MMP11 – matrix metallopeptidase 11 (stromelysin 3)
Hs.50081.1.S1_3p_a_at	1.36	5.47 × 10^-3^	KIAA1199 – KIAA1199
g11641276_3p_s_at	1.24	3.71 × 10^-2^	PDE4DIP – phosphodiesterase 4D interacting protein (myomegalin)
Hs.169517.0.S1_3p_a_at	1.16	5.72 × 10^-3^	ALDH1B1 – aldehyde dehydrogenase 1 family, member B1
Hs.98523.0.A1_3p_at	1.13	5.32 × 10^-3^	FAT3 – FAT tumor suppressor homolog 3 (Drosophila)
g10938018_3p_at	1.04	1.74 × 10^-2^	EPYC – epiphycan
Hs2.350890.1.S1_3p_s_at	1.03	3.72 × 10^-2^	GABRB2 – gamma-aminobutyric acid (GABA) A receptor, beta 2
g4507922_3p_at	0.98	1.70 × 10^-2^	WISP2 – WNT1 inducible signaling pathway protein 2
g11342665_3p_at	0.98	4.53 × 10^-2^	MMP2 – matrix metallopeptidase 2 (gelatinase A, 72kDa gelatinase, 72 kDa type IV collagenase)
g13124890_3p_a_at	0.93	3.28 × 10^-2^	GALNT1 – UDP-*N*-acetyl-alpha-D-galactosamine:polypeptide *N*-acetylgalactosaminyltransferase 1 (GalNAc-T1)
Hs.98523.0.A1_3p_x_at	0.87	3.15 × 10^-2^	FAT3 – FAT tumor suppressor homolog 3 (Drosophila)
Hs.238532.0.A1_3p_at	0.76	3.71 × 10^-2^	GALNTL2 – UDP-*N*-acetyl-alpha-D-galactosamine:polypeptide *N*-acetylgalactosaminyltransferase-like 2
Hs.42927.0.S1_3p_at	0.75	3.39 × 10^-2^	ANTXR1 – anthrax toxin receptor 1
Hs2.359399.1.S1_3p_at	0.74	3.71 × 10^-2^	LOC285758 – hypothetical protein LOC285758
Hs.235795.0.A1_3p_at	0.74	1.38 × 10^-2^	-
Hs2.46679.2.S1_3p_s_at	0.66	2.11 × 10^-3^	-
g469044_3p_a_at	0.60	3.22 × 10^-2^	CNTN1 – contactin 1
g4758607_3p_at	0.53	1.20 × 10^-2^	-
Hs.288553.0.S1_3p_s_at	0.52	2.82 × 10^-2^	-
200661_3p_at	0.51	4.16 × 10^-2^	CTSA – cathepsin A
Hs.2399.1.S1_3p_s_at	0.51	4.48 × 10^-2^	MMP14 – matrix metallopeptidase 14 (membrane-inserted)
Hs.98183.0.A1_3p_at	0.48	8.58 × 10^-3^	RSPO4 – R-spondin family, member 4
208756_3p_at	0.48	2.28 × 10^-2^	EIF3I – eukaryotic translation initiation factor 3, subunit I
Hs.162647.0.S1_3p_at	0.48	2.69 × 10^-2^	DKFZP547L112 – hypothetical protein DKFZp547L112
Hs.22968.0.S1_3p_a_at	-2.00	8.37 × 10^-6^	FLT1 – fms-related tyrosine kinase 1 (vascular endothelial growth factor/vascular permeability factor receptor)
g11545907_3p_at	-2.03	2.68 × 10^-5^	ELTD1 – EGF, latrophilin and seven transmembrane domain containing 1
g5032094_3p_at	-2.03	3.64 × 10^-7^	SLCO2A1 – solute carrier organic anion transporter family, member 2A1
Hs.8707.0.S1_3p_at	-2.07	1.29 × 10^-5^	HECW2 – HECT, C2 and WW domain containing E3 ubiquitin protein ligase 2
Hs.134970.0.S1_3p_a_at	-2.07	6.66 × 10^-3^	KIF26A – kinesin family member 26A
Hs2.420404.1.S1_3p_at	-2.08	7.70 × 10^-6^	PELO – pelota homolog (Drosophila)
g4504850_3p_a_at	-2.11	3.62 × 10^-2^	KCNK5 – potassium channel, subfamily K, member 5
Hs.288681.0.S1_3p_at	-2.13	4.98 × 10^-4^	THSD7A – thrombospondin, type I, domain containing 7A
g4557546_3p_at	-2.15	1.58 × 10^-3^	EDNRB – endothelin receptor type B
g11321596_3p_at	-2.19	1.64 × 10^-3^	KDR – kinase insert domain receptor (a type III receptor tyrosine kinase)
Hs.124675.0.A1_3p_at	-2.21	2.21 × 10^-4^	GIMAP7 – GTPase, IMAP family member 7
Hs.211388.0.S1_3p_at	-2.24	5.72 × 10^-3^	RUNDC3B – RUN domain containing 3B
g4885556_3p_at	-2.25	2.13 × 10^-3^	PODXL – podocalyxin-like
Hs.26530.0.S2_3p_at	-2.30	1.60 × 10^-3^	SDPR – serum deprivation response (phosphatidylserine binding protein)
g13518036_3p_a_at	-2.41	7.19 × 10^-3^	MATN2 – matrilin 2
Hs.102415.0.S1_3p_at	-2.43	2.67 × 10^-8^	EMCN – endomucin
Hs.61935.0.S1_3p_at	-2.45	2.68 × 10^-5^	PCDH17 – protocadherin 17
g8547214_3p_at	-2.46	3.74 × 10^-5^	EMCN – endomucin
g4520327_3p_at	-2.48	2.67 × 10^-3^	IL33 – interleukin 33
Hs.10587.0.S1_3p_at	-2.66	2.29 × 10^-3^	DMN – desmuslin
g3644039_3p_a_at	-2.67	1.43 × 10^-2^	TP63 – tumor protein p63
Hs.78344.1.S2_3p_a_at	-2.87	2.13 × 10^-3^	MYH11 – myosin, heavy chain 11, smooth muscle
g6580814_3p_s_at	-2.93	8.90 × 10^-5^	INMT – indolethylamine *N*-methyltransferase
Hs.173560.0.S1_3p_at	-2.94	3.58 × 10^-2^	ODZ2 – odz, odd Oz/ten-m homolog 2 (Drosophila)
g4506870_3p_at	-3.23	1.80 × 10^-3^	SELE – selectin E (endothelial adhesion molecule 1)

### Stromal gene expression signature associated with tumor grade

**Figure 5:**
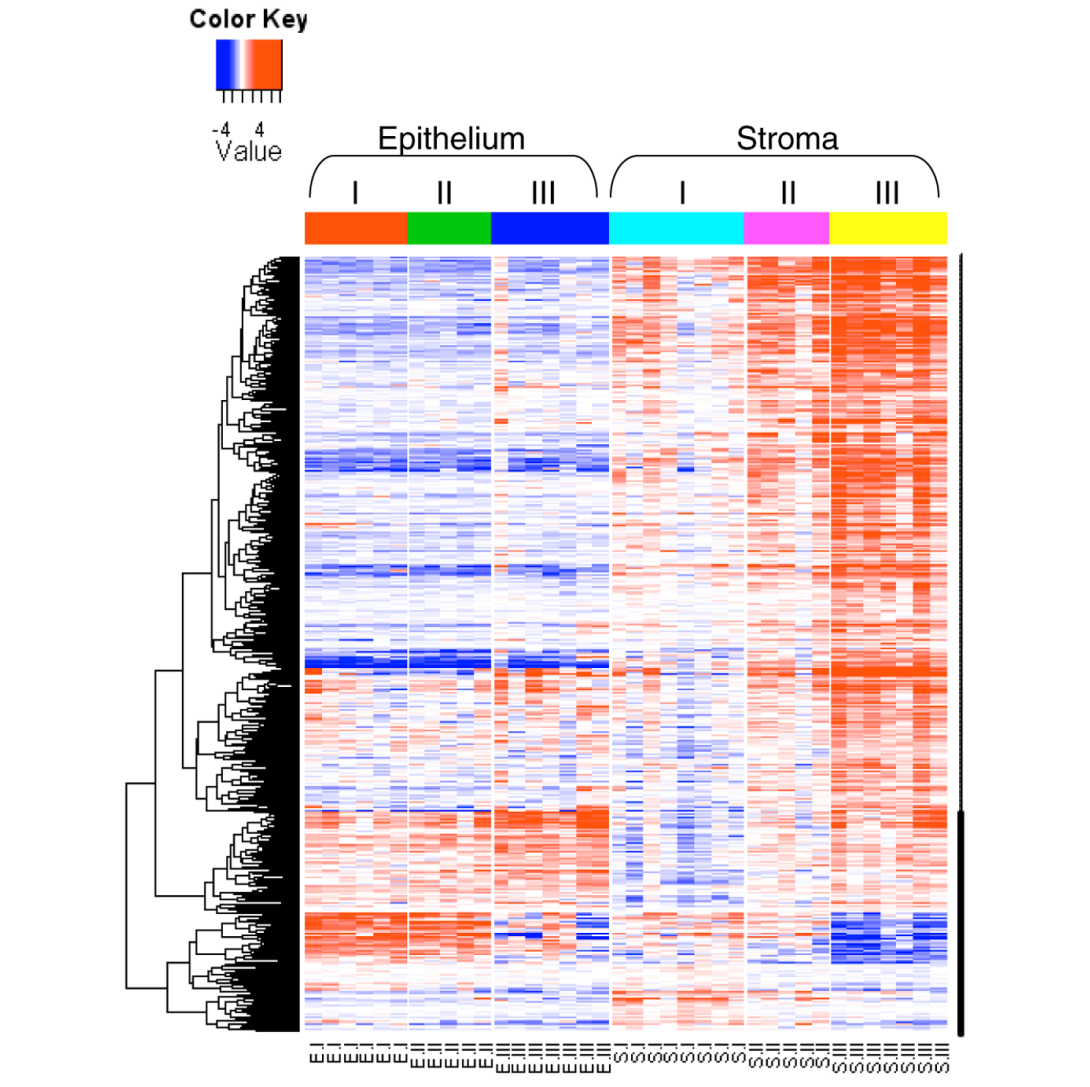
Heatmap of gene expression signature correlated with tumor grade in the stroma. Comparison of grade III tumors with grade I tumors identified 526 upregulated genes and 94 downregulated genes in grade III stroma. Data shown are log_2_(fold change) relative to the median expression level across all samples. Genes in rows were hierarchically clustered, and samples in columns were arranged by sample type. E, epithelium; S, stroma.

We have previously shown that tumor grade is associated with a strong gene expression signature in malignant breast epithelial cells [9]. We therefore examined whether a similar signature also exists in the tumor stroma. Comparing grade I (*n* = 8) and grade III (*n* = 7) tumor-associated stroma samples (DCIS-S and IDC-S), we identified 526 upregulated genes and 94 downregulated genes in grade III samples (Figure 5; see also Additional data file 2). The gene set enrichment analysis indicated that the tumor stroma in grade III tumors were associated with a strong immune response signature (interferon signaling, activation of leukocytes and T cells) and with increased mitotic activity (Table 7).

**Table 7:** Top 20 gene sets enriched in grade III-associated stroma

Name	Size (number of genes)	Normalized enrichment score	False discovery rate *q *value
CELLULAR_DEFENSE_RESPONSE	52	2.31	0
IMMUNE_RESPONSE	220	2.17	0
IMMUNE_SYSTEM_PROCESS	312	2.16	0
T_CELL_ACTIVATION	42	2.14	0
LEUKOCYTE_ACTIVATION	67	2.09	0
JAK_STAT_CASCADE	28	2.05	6.82 × 10^-4^
LYMPHOCYTE_ACTIVATION	59	2.05	5.85 × 10^-4^
CELL_ACTIVATION	73	2.04	5.12 × 10^-4^
M_PHASE_OF_MITOTIC_CELL_CYCLE	78	2.04	4.55 × 10^-4^
RESPONSE_TO_VIRUS	48	2.04	5.12 × 10^-4^
SPINDLE	39	2.03	5.60 × 10^-4^
MITOSIS	75	2.02	5.99 × 10^-4^
INTERLEUKIN_RECEPTOR_ACTIVITY	20	2.01	6.33 × 10^-4^
POSITIVE_REGULATION_OF_IMMUNE_RESPONSE	28	2.00	7.35 × 10^-4^
REGULATION_OF_IMMUNE_SYSTEM_PROCESS	66	1.99	7.54 × 10^-4^
POSITIVE_REGULATION_OF_IMMUNE_SYSTEM_PROCESS	50	1.99	7.07 × 10^-4^
RESPONSE_TO_BIOTIC_STIMULUS	112	1.99	6.65 × 10^-4^
REGULATION_OF_I_KAPPAB_KINASE_NF_KAPPAB_CASCADE	89	1.99	6.85 × 10^-4^
MRNA_PROCESSING_GO_0006397	67	1.97	0.001135
RESPONSE_TO_OTHER_ORGANISM	76	1.96	0.001282

### Validation of selected differentially expressed genes

**Figure 6:**
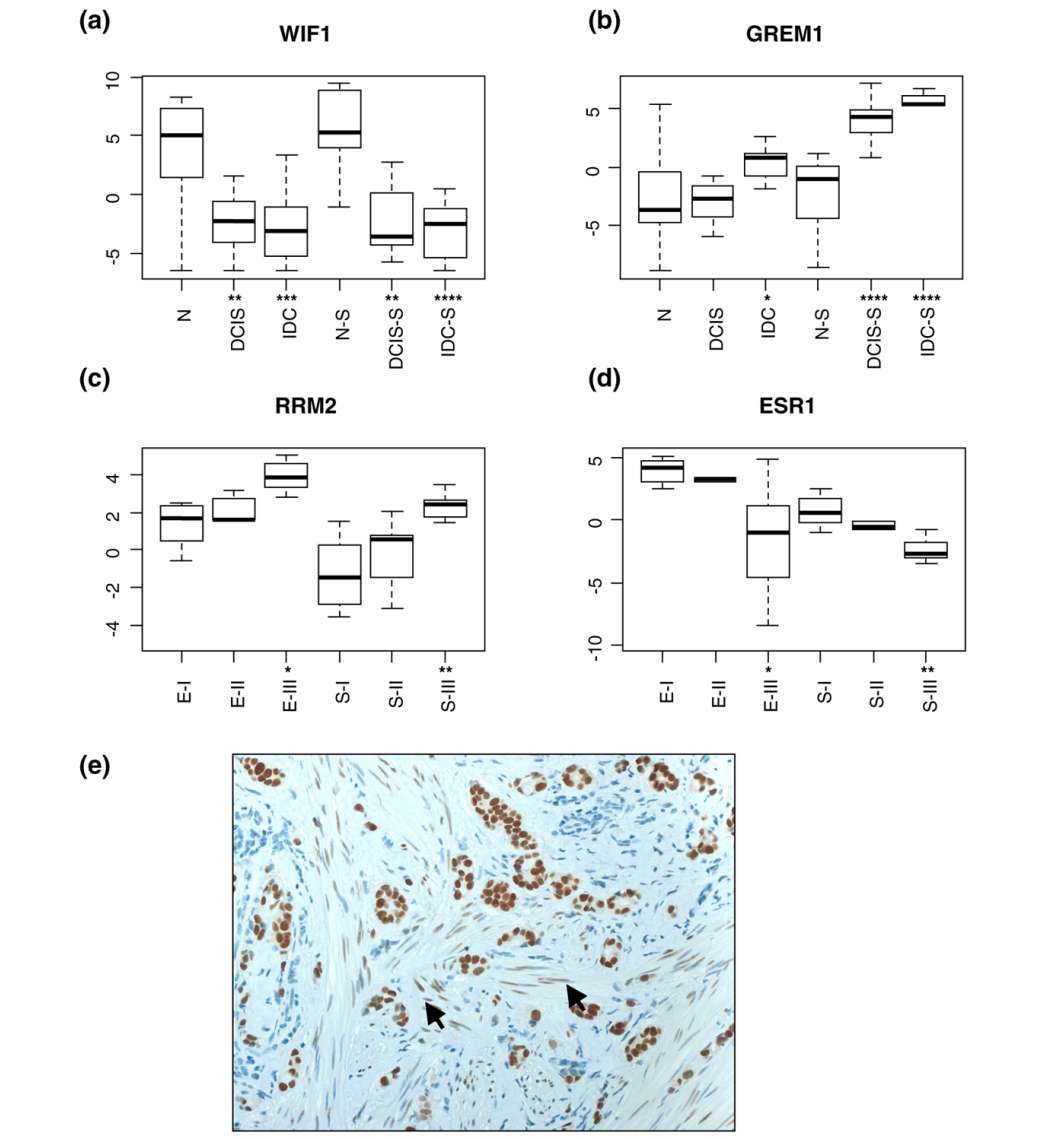
Validation of selected genes. **(a) **to **(d) **Boxplots of relative gene expression by quantitative real-time PCR in ductal carcinoma *in situ *(DCIS), invasive ductal carcinoma (IDC), ductal carcinoma *in situ*-associated stroma (DCIS-S) and invasive ductal carcinoma-associated stroma (IDC-S). (a) and (b) Reference groups were the normal components (N, normal breast epithelium; N-S, normal stromal compartment). (c) and (d) Reference groups were grade I (EI, epithelium; SI, stroma). *y *axis, cycling threshold values relative to the median value for the entire series. Statistically significant differences by Wilcoxon rank sum test: *P < 0.05, **P < 0.01, ***P < 0.001, ****P < 0.0001.**(e) **Immunostaining of an estrogen-receptor-positive breast cancer. Arrows point to positive staining in stromal fibroblasts.

We next used quantitative real-time PCR to validate selected genes differentially expressed in the various comparisons presented above. Quantitative real-time PCR analysis of the same samples as used in the microarray analysis confirmed the marked downregulation of WIF1 in both neoplastic epithelium and tumor stroma (Figure 6a) and the marked upregulation of GREM1 in both DCIS-associated and IDC-associated stroma (Figure 6b). In addition, two representative genes (ESR1, estrogen receptor alpha; and RRM2, ribonucleotide reductase M2 subunit) differentially expressed in the stroma between grade III and grade I tumors (see Additional data file 2) were also confirmed by quantitative real-time PCR. In both the epithelium and stroma, RRM2, a cell proliferation marker, was more highly expressed in grade III tumors (Figure 6c), whereas ESR1 was more highly expressed in grade I tumors (Figure 6d). Although expression of estrogen receptor alpha is thought to be restricted to the tumor epithelial cells in human breast cancer [24], we confirmed the low but detectable levels of estrogen receptor alpha expression in stromal fibroblasts by immunohistochemical staining (Figure 6e).

## Discussion

Exploratory genome-wide analysis of the tumor microenvironment in breast cancer has been limited to date. Using serial analysis of gene expression coupled with antibody-based *ex vivo *tissue fractionation, Allinen and colleagues identified a limited set of 417 cell-type-specific genes among the most prominent cell types in breast cancer (epithelial, myoepithelial, and endothelial cells, fibroblasts, and leukocytes) [7]. Finak and colleagues more recently obtained gene expression profiles of both epithelial and stromal compartments from the same tumor biopsy via LCM [25]. These workers only analyzed the morphologically normal epithelium and normal stroma, however, leaving the gene expression changes in the tumor-activated stroma unexplored. Our work therefore provides the first comprehensive comparative analysis of *in vivo *gene expression changes in the tumor epithelium and its stromal microenvironment during breast cancer progression from normal to DCIS to IDC.

We observed extensive gene expression changes in the stroma associated with DCIS and IDC, suggesting that tumor-adjacent stroma coevolves with the tumor epithelium, even before tumor invasion occurs. These alterations included many components of the extracellular matrix and the extracellular-matrix-remodeling matrix metalloproteases. Increased mitotic gene expression occurred both in the malignant epithelium and adjacent stroma, which may reflect the often observed desmoplastic reaction around the tumor cells. Expression of cytoplasmic ribosomal proteins was generally decreased in both compartments during cancer progression. While this result may seem paradoxical in that increased protein synthesis is considered a hallmark of cancer, it is supported by several different lines of studies. First, decreased expression of many ribosomal proteins has also been observed in colorectal cancer compared with normal mucosal epithelium [26]. Secondly, many ribosomal protein genes have been found to be haploinsufficient tumor suppressors in zebrafish [27]. Thirdly, the oncogenic activity of c-Myc is inhibited by the ribosomal protein L11, and inactivation of the L11 gene by small interfering RNA increases c-Myc-induced transcription and cell proliferation [28].

The mechanism by which ribosomal proteins contribute to tumorigenesis is unknown. Decreased expression of ribosomal proteins in cancer may reflect a qualitative change in ribosomal structure, which may allow differential translation of gene products required for rapid tumor growth. Alternatively, it may reflect some unknown nonribosomal functions by these proteins. In contrast to the decreased expression of these cytoplasmic ribosomal protein genes, we observed increased expression of a number of mitochondrial ribosomal protein genes in both the tumor epithelium and the stroma. The human mitochondrial ribosomes are responsible for the production of several key proteins in bioenergetics including subunits of the ATP synthase. Given the importance of mitochondria in cancer [29,30], our novel finding suggests that the mitochondrial ribosome may be a potential therapeutic target and thus warrants further study.

The top differentially expressed genes between tumor-associated stroma and the adjacent normal stroma included several signaling molecules known to be important for tumorigenesis. Two antagonists of WNT receptor signaling, WIF1 and SFRP1, were consistently downregulated both in the tumor epithelium and stroma. The WNT signaling pathway plays an important role in development and tissue homeostasis, and its aberrant activation by loss of expression WIF1 or SFRP1 has been shown to be an important early event in breast cancer progression [31-33]. Two transforming growth factor beta superfamily members (GREM1 and INHBA) are strongly induced in the tumor-associated stroma. GREM1 is a bone morphogenetic protein antagonist, and it is overexpressed in cancer-associated stromal cells in many solid tumors [34]. It has been hypothesized that bone morphogenetic proteins and bone morphogenetic protein antagonists may play opposing roles in the maintenance of a niche of self-renewing stem cells, with bone morphogenetic protein antagonists such as GREM1 blocking cell differentiation [34]. WNT3A was recently demonstrated in human fibroblasts to markedly increase the expression of GREM2, a close paralog of GREM1 – raising the possibility that the significant downregulation of WNT antagonists (WIF1 and SFRP1) and upregulation of GREM1 in the stroma [35] we observed here may be functionally linked.

INHBA is the gene for the beta A subunit of inhibin and activin, which are pleiotropic growth factors regulating the growth and differentiation of many cell types via autocrine and paracrine mechanisms [36]. Although its role in breast cancer remains unclear, circulating levels of INHBA has been shown to be higher in breast cancer patients with bone metastasis [37]. These signaling molecules could serve as key messengers between the tumor and its microenvironment, as shown for CXCL12 and CXCL14, which are overexpressed in tumor-associated myoepithelial cells and myofibroblasts [6,7,38]. We note that in our dataset, however, CXCL12 and CXCL14 were also expressed in normal stroma. This discrepancy could be due to the fact that Allinen and colleagues used purified stromal cell types [7] and we used the whole stroma compartment in our study.

A watershed event in breast cancer progression is the invasion of tumor cells into the stromal compartment. The only morphological diagnostic criterion distinguishing DCIS from IDC is the association of DCIS with a complete basement membrane. Understanding the molecular events that drive the DCIS-IDC transition has been of great interest. We have previously shown [9], and confirm in the present study, that the malignant epithelium of DCIS and IDC are very similar without significant differences at the transcriptome level. This conclusion is supported by the recent demonstration that MCFDCIS cells, a cell line model for DCIS, make the DCIS-IDC transition spontaneously without further molecular changes in the malignant epithelial cells themselves [39]. Instead, this transition is driven by fibroblasts and blocked by myoepithelial cells.

In the present article we demonstrated that the stromal compartment is associated with a relatively small number of significant changes accompanying the DCIS-IDC transition. In particular, several matrix metalloproteases (MMP2, MMP11 and MMP14) showed significantly increased expression in IDC-associated stroma. MMP14, a membrane-type matrix metalloprotease, can activate MMP2 protease activity, which degrades type IV collagen, the major structural component of the basement membrane [40,41]. MMP11 has recently been shown to exhibit protease activity towards type VI collagen and to promote tumor progression [42]. MMP11 has been shown to be differentially expressed in IDC relative to DCIS in two other studies. Schuetz and colleagues conducted a study similar to ours, using LCM and microarrays to profile the epithelium of patient-matched DCIS and IDC, and found MMP11 to be upregulated in IDC relative to DCIS [43]. Their result differs from ours, however, in that we observed upregulation of MMP11 in the IDC-associated stroma but not in the epithelium. A stromal origin of MMP11 expression had been established previously [44]. The result by Schuetz and coworkers might be due to contaminating nonepithelial cells in their LCM samples, a possibility acknowledged by these authors [43]. In another study, Hannemann and colleagues identified a gene expression signature including MMP11 to be able to distinguish IDC from DCIS [45]. Since no microdissection was performed in that study, the gene expression profiles they obtained were from mixtures of tumor epithelium and stroma. Nevertheless, our results together with these other studies support the notion that stroma-produced matrix metalloproteases may be key players driving the DCIS-IDC transition.

Finally, we showed that – like the epithelial compartment [9] – tumor stroma also exhibited a robust gene expression signature correlating with the histological tumor grade. These genes are primarily involved in immune response and cell-cycle progression. The association of an immune response signature with the more aggressive high-grade tumors is seemingly paradoxical. The interactions between tumor cells and the various immune cells are complex, however, ranging from tumor growth-suppressing effects to tumor growth-promoting effects [46-48]. Perhaps the immune response signature associated with high-grade tumors represents the escape phase [48], when the cancer cells become resistant to immune attack and hijack the abundant cytokines and chemokines made by the immune cells to grow, invade and spread to distant organs.

## Conclusions

The present study provides the first comparative analysis of the *in situ *gene expression profiles of patient-matched normal and neoplastic breast epithelial and stromal compartments of both preinvasive and invasive stages of human breast cancer progression. This study of the breast cancer microenvironment at the transcriptome level and previous studies at the genomic [49,50] and epigenetic [51,52] levels support the view that the tumor microenvironment is an important co-conspirator rather than a passive bystander during tumorigenesis. Molecular alterations within the stroma offer novel avenues for therapeutic interventions and disease prognosis [53]. This gene expression dataset of carefully procured *in situ *tumor epithelium and stroma should be a timely and valuable addition to the resources for the breast cancer research community.

## Abbreviations

CXCL: chemokine (C-X-C motif) ligand; DCIS: ductal carcinoma *in situ*; DCIS-S: DCIS-associated stroma; GREM1: gremlin 1; IDC: invasive ductal carcinoma; IDC-S: IDC-associated stroma; INHBA: inhibin beta A; LCM: laser capture microdissection; PCR: polymerase chain reaction; SFRP1: secreted frizzled-related protein 1; WIF1: WNT inhibitory factor 1.

## Competing interests

XJM and ME are employees of BioTheranostics, Inc. ME, X-JM and DCS are named inventors on a patent application relating to the contents of the manuscript.

## Authors' contributions

DCS, XJM and ME conceived of the study, participated in its design and coordination, and helped draft the manuscript. DCS and SD performed microdissection and RNA extraction. XJM and ME performed the microarray experiments and microarray data analysis. ER performed the real-time PCR assays. All authors have read and approved the final manuscript.

## Supplementary Material

Additional file 1 An Excel file containing a table that lists the genes differentially expressed between DCIS-S and IDC-S. Click here for file

Additional file 2 An Excel file containing a table that lists the genes differentially expressed between grade III and grade I samples. Click here for file
